# Computational Design of Phosphotriesterase Improves V‐Agent Degradation Efficiency

**DOI:** 10.1002/open.202300263

**Published:** 2024-03-01

**Authors:** Jacob Kronenberg, Stanley Chu, Andrew Olsen, Dustin Britton, Leif Halvorsen, Shengbo Guo, Ashwitha Lakshmi, Jason Chen, Maria Jinu Kulapurathazhe, Cetara A. Baker, Benjamin C. Wadsworth, Cynthia J. Van Acker, John G. Lehman, Tamara C. Otto, P. Douglas Renfrew, Richard Bonneau, Jin Kim Montclare

**Affiliations:** ^1^ Department of Chemical and Biomolecular Engineering New York University Tandon School of Engineering Brooklyn New York United States; ^2^ Center for Genomics and Systems Biology New York University New York New York United States; ^3^ Center for Computational Biology Flatiron Institute New York New York United States; ^4^ Medical Toxicology Research Division U.S. Army Medical Research Institute of Chemical Defense Aberdeen Proving Ground Maryland United States; ^5^ Department of Biomaterials New York University College of Dentistry New York New York United States; ^6^ Department of Radiology New York University Grossman School of Medicine New York New York United States; ^7^ Department of Biomedical Engineering New York University Tandon School of Engineering Brooklyn New York United States; ^8^ Department of Chemistry New York University New York New York United States

**Keywords:** enzyme engineering, organophosphates, nerve agents, computational design, nanoscavengers

## Abstract

Organophosphates (OPs) are a class of neurotoxic acetylcholinesterase inhibitors including widely used pesticides as well as nerve agents such as VX and VR. Current treatment of these toxins relies on reactivating acetylcholinesterase, which remains ineffective. Enzymatic scavengers are of interest for their ability to degrade OPs systemically before they reach their target. Here we describe a library of computationally designed variants of phosphotriesterase (PTE), an enzyme that is known to break down OPs. The mutations G208D, F104A, K77A, A80V, H254G, and I274N broadly improve catalytic efficiency of VX and VR hydrolysis without impacting the structure of the enzyme. The mutation I106 A improves catalysis of VR and L271E abolishes activity, likely due to disruptions of PTE's structure. This study elucidates the importance of these residues and contributes to the design of enzymatic OP scavengers with improved efficiency.

## Graphical abstract

Organophosphates (OPs) are potent neurotoxins whose current remedies are not very effective. Here we design and characterize variants of the enzyme phosphotriesterase, which can degrade OPs. We report mutations improving catalytic efficiency between 2‐ and 5‐fold and confirm folding and stability of the resulting variants. These findings are a step towards improved OP bioscavengers.

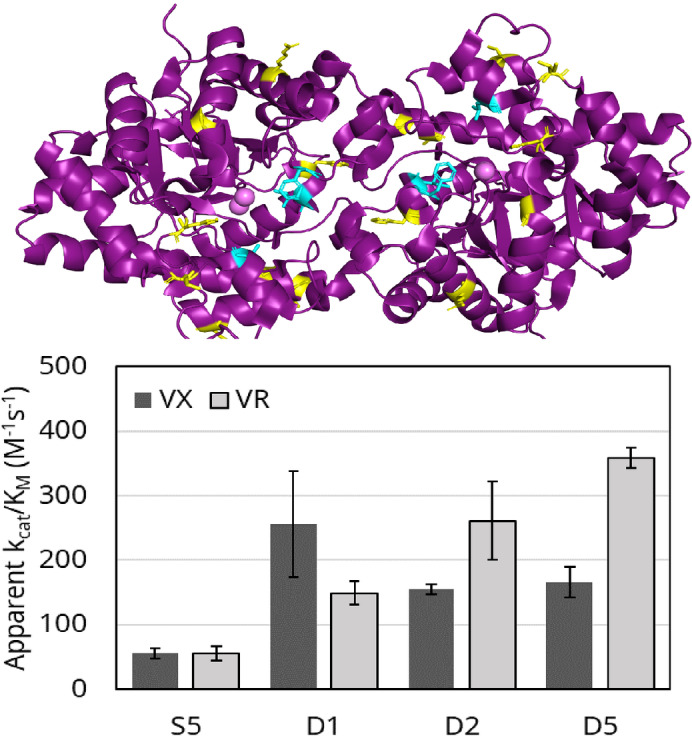

## Introduction

Organophosphates (OPs) are a class of compounds including commonly used pesticides as well as highly toxic nerve agents.^[1]^ They inhibit the enzyme acetylcholinesterase, which results in a buildup of acetylcholine at the nerve junctions leading to a cholinergic crisis. Current treatment of these nerve agents relies on administration of oximes, which can sometimes reactivate acetylcholinesterase but do not neutralize the OPs present.^[2]^ It is therefore important to develop new strategies to counteract organophosphates.

The enzyme phosphotriesterase (PTE, also known as organophosphorus hydrolase) is able to break down OPs.^[3]^ While PTE is effective against paraoxon, believed to be its native substrate, its application against other OPs has been plagued by low efficacy and low solubility. Early work on PTE has used directed evolution to engineer PTE from *Brevundimonas diminuta* into the S5 variant (PTE‐S5), with three point mutations that improve soluble expression.^[4]^ More recent work has engineered PTE to work effectively against a wide variety of organophosphate substrates including sarin and VX,^[5]^ but work on highly toxic organophosphates is often limited by safety concerns or performed on less toxic OP analogues, leading to less accurate results.^[6]^ Moreover, wild‐type PTE has been shown to have increased hydrolysis activity on R enantiomers, known to be less toxic than S enantiomers of VX and VR.^[7]^ Thus, significant work has been focused on increasing the enantioselectivity of PTE.^[8]^

**Scheme 1:**
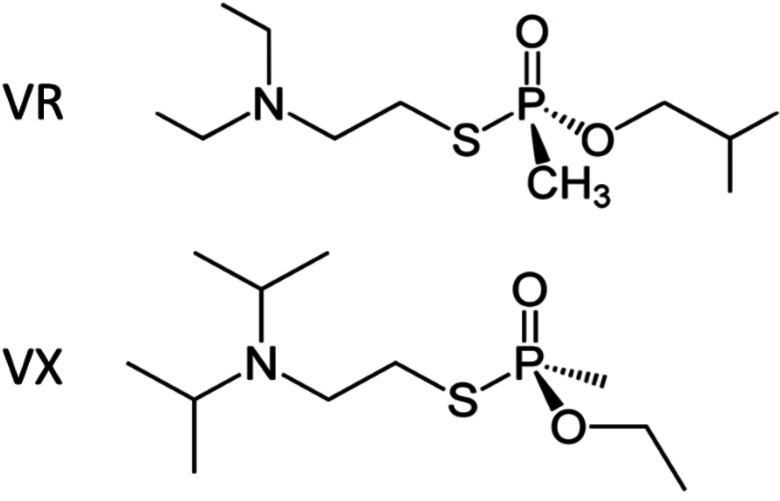
The structures of OP nerve agents VR and VX. The S enantiomers are shown, which are more toxic than the R enantiomers.

**Figure 1:**
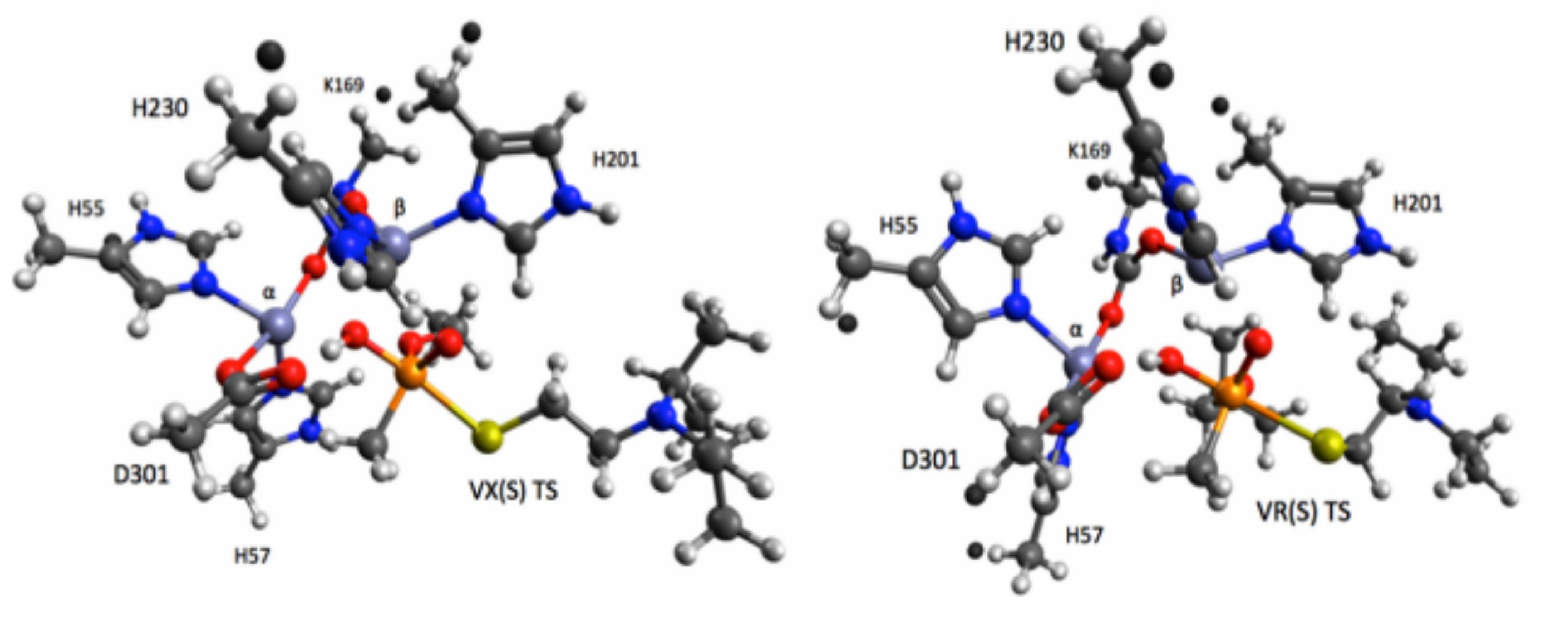
Transition states of the S enantiomers of VX (left) and VR (right) docked into the active site of PTE.

In this work, we used computational strategies to design a library of PTE variants to break down two organophosphorus nerve agents: VX and VR using docking of S enantiomers (Scheme 1, Figure 1). We modeled enzyme variants using Rosetta, a software suite for macromolecular design,^[9]^ to select candidates for experimental screening. We synthesized six variants, measured their efficacy against VX and VR, and performed structural and thermal characterization in the Co^2+^ form. We specifically explore the Co^2+^ form due to its higher activty than forms with other cofactors.^[10]^ Moreover, where Zn^2+^ is more abundant in the human body,^[11]^ Co^2+^ remains relevant for envionrmental detoxification conditions compared to Zn^2+^, which may be more suitable for *in vivo* applications. Three of the variants exhibit improved activity against VX and VR, which gives important insight to the design of future enzymes for OP detoxification.

## Results/Discussion

### Design and Modelling of Variants

Rossetta macromolecular modleing suite was employed to assess the binding energies of variants of PTE‐S5 based on mutations to a model of PTE‐S5 studied previously^[12]^ (**SI**). Six designs with the top scoring binding energies were selected and numbered PTE‐D1 through PTE‐D6 (Table 1). All six shared several mutations including G208D (reversion of the G in PTE‐S5 to the D in the wildtype),^[4]^ F104A (previously found to resolve a steric clash at the dimer interface),^[13]^ K77A, A80V, H254G, and I274N. PTE‐D1 differs from PTE‐S5 only by these six mutations. The remaining five variants have different combinations of mutations near the active site: I106A is present in PTE‐D2, PTE‐D4, and PTE‐D5, F132E is present in PTE‐D5 and PTE‐D6, and L271E is present in PTE‐D3, PTE‐D4, and PTE‐D6. All variants had lower Rosetta energy scores than PTE‐S5, indicating increased stability (Table 1). All had negative binding scores to hydrolysis transition states of the S enantiomers of both VX and VR (Figure 1), suggesting that they will stabilize the transition state and catalyze hydrolysis. In particular, PTE‐D2 and PTE‐D5 had significantly lower binding scores than PTE‐S5 for VR, suggesting they may be particularly active against that substrate.

**Table 1:** Point mutations present in PTE‐S5 and each of seven new PTE variants along with their Rosetta scores and binding scores with VX and VR. An (x) in a column indicates that mutation is present in the corresponding variant.

	I106A	F132E	L271E	Full Score (kJ/mol)	VX Binding (kJ/mol)	VR Binding (kJ/mol)
PTE‐S5				−2130	−4.36	−17.08
PTE‐D1				−2175	−4.03	−15.79
PTE‐D2	x			−2170	−3.99	−18.22
PTE‐D3			x	−2140	−2.05	−14.00
PTE‐D4	x		x	−2159	−1.50	−15.72
PTE‐D5	x	x		−2150	−3.89	−17.86
PTE‐D6		x	x	−2150	−2.36	−13.72

### Conformational Analysis and Stability

**Figure 2:**
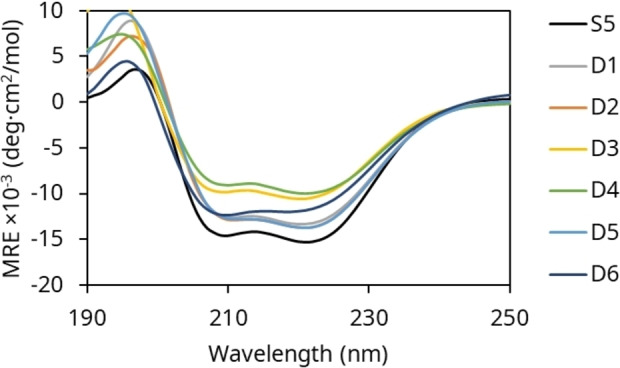
CD spectra of PTE variants showing preservation of α‐helical structure across variants with slight changes in PTE‐D3, PTE‐D4 and PTE‐D6.

To assess the impact of the mutations on protein structure, circular dichroism (CD) spectroscopy was carried out (Figure 2). CD spectra of PTE‐S5 have minima of −14200±3600 deg ⋅ cm^2^/mol and −15200±3800 deg ⋅ cm^2^/mol at 208 nm and 222 nm respectively, indicating α‐helical structure (Table 2).^[12]^ PTE‐D1, PTE‐D2, and PTE‐D5 are not significantly different from PTE‐S5. PTE‐D3 and PTE‐D4 have smaller minima of −10400±800 deg ⋅ cm^2^/mol and −10000±1700 deg ⋅ cm^2^/mol at 222 nm and −9700±800 deg ⋅ cm^2^/mol and −8900±1800 deg ⋅ cm^2^/mol at 208 nm suggesting weaker structure. All of the variants studied have θ_222_/θ_208_ values of 1.0 to 1.1, which is consistent with past measurements and with the TIM barrel structure of PTE.^[12]^

**Table 2:** Circular dichroism spectroscopy results of PTE variants at 25 °C including mean residual ellipticity at 208 nm and 222 nm, the ratio between those peak heights, and the approximate structural composition as determined by BeStSel.

	θ208 (kdeg ⋅ cm^2^/mol)	θ222 (kdeg ⋅ cm^2^/mol)	θ222/θ208	% α‐helix	% β‐sheet	% other
PTE‐S5	−14.2±3.8	−15.2±3.8	1.1±0.4	38.7±4.4	21.0±2.2	40.4±4.2
PTE‐D1	−12.0±0.4	−13.2±0.7	1.1±0.1	30.9±1.5	19.0±2.1	50.1±3.4
PTE‐D2	−12.1±0.9	−13.7±1.1	1.1±0.1	29.6±3.1	22.2±2.1	48.2±3.2
PTE‐D3	−9.7±0.8	−10.4±0.8	1.1±0.1	24.1±1.9	18.8±5.2	57.2±4.0
PTE‐D4	−8.9±1.8	−10.0±1.7	1.1±0.3	23.4±4.0	19.0±2.0	57.6±4.0
PTE‐D5	−12.0±1.8	−13.7±2.3	1.1±0.3	33.4±1.9	20.0±5.1	46.6±6.6
PTE‐D6	−12.1±1.0	−11.7±0.9	1.0±0.1	26.5±0.8	19.4±1.4	54.1±2.3

BeStSel was used to approximate the contribution of each secondary structure class to the spectrum of each variant (Table 2).^[14]^ PTE‐S5 had 38.7±4.4 % α‐helical and 21.0±2.2 % β‐sheet content, which is consistent with its TIM barrel fold. PTE‐D3, PTE‐D4 and PTE‐D6 demonstrated a decrease in α‐helical content and an increase in other/random coil content, but no change in β‐sheet content relative to PTE‐S5. Other variants did not exhibit a significant change in secondary structure fractions relative to PTE‐S5.

**Figure 3:**
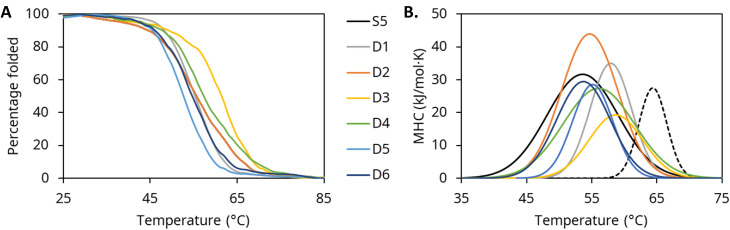
A. Melting curves of the seven PTE variants as calculated by decrease in CD signal at 222 nm corresponding to loss of α‐helical structure; B. DSC thermograms showing the melting temperatures of each of the PTE variants (solid lines) as well as the additional thermal transition present in S5 corresponding to the disassociation of the dimer state (dashed line).

Thermal characterization was also carried out with differential scanning calorimetry (DSC). DSC thermograms (Figure 3) agreed broadly with CD temperature scans. All variants showed melting temperatures (T_M_) close to PTE‐S5’s T_M_ of 55 °C (Table 3). As expected, PTE‐S5 exhibited two transitions with a lower T_M_ 54.5±0.5 °C and an upper T_M_ of 64.5±0.1 °C, consistent with previous results.^[15]^ Only the first transition is visible by CD since the second one does not significantly change the secondary structure. While PTE‐S5 possessed a second transition, it was absent from other variants. This suggested that the mutations present in all of the tested variants likely prevented the protein chains from remaining associated after the loss of secondary structure, behavior that has been attested in other PTE variants.^[13]^ All variants other than PTE‐D3 and PTE‐D4 exhibited agreement between T_M_ values measured by CD and DSC, but PTE‐D3 and PTE‐D4 showed slightly higher T_M_ values by CD. Since CD measures T_M_ indirectly through loss of α‐helicity,^[16]^ this discrepancy could be due to other structures melting at a lower temperature being observed by DSC before the α‐helices measured by CD.

**Table 3:** Melting temperatures of PTE variants as measured by DSC and CD spectroscopy. PTE‐S5 showed two thermal transitions with DSC, whereas all other variants showed a single transition with both techniques.

	DSC T_M_ (°C)	CD T_M_ (°C)
PTE‐S5	54.5±0.5	55.8±2.3
	64.5±0.1	
PTE‐D1	56.7±0.7	55.3±1.3
PTE‐D2	55.0±0.1	54.5±0.4
PTE‐D3	56.5±1.1	60.9±0.5
PTE‐D4	54.9±0.7	57.8±1.1
PTE‐D5	54.0±0.8	52.7±0.7
PTE‐D6	54.1±0.3	54.8±1.5

### OP Hydrolysis Kinetics

**Figure 4:**
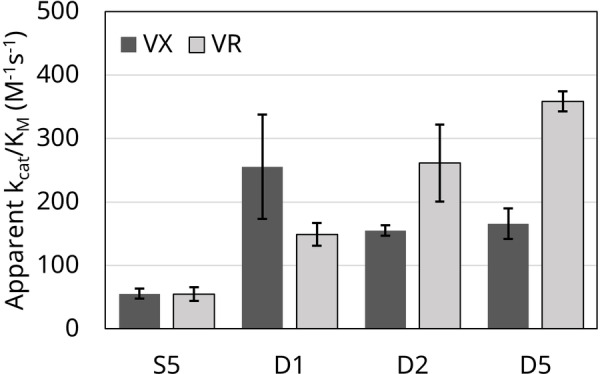
Apparent catalytic efficiency of PTE‐S5, PTE‐D1, PTE‐D2 and PTE‐D5 against VX and VR. PTE‐D3, PTE‐D4, and PTE‐D6 had no detectable activity.

Hydrolysis was measured in crude lysate containing each variant. In our preliminary assessment, we also found that PTE‐D1, PTE‐D2, and PTE‐D5 were active against paraoxon while others were not, where we further focused on quantifying hydrolysis of VX and VR for the scope of this work. Along with PTE‐S5, variants PTE‐D1, PTE‐D2 and PTE‐D5 exhibited efficacy against both VX and VR using racemic mixtures, whereas variants PTE‐D3, PTE‐D4, and PTE‐D6 had no detectable activity (Table 4). Because expression levels may differ between variants, lysate assays cannot give qualitative measures of kinetics, so the functional variants PTE‐D1, PTE‐D2, and PTE‐D5 were selected for purification and further characterization (Figure 4, Table 4). At the maximum allowable concentrations of VX and VR, no saturation occurred, indicating that K_M_>[S]_max_. Hence, k_cat_ and K_M_ could not be directly calculated and instead, apparent k_cat_/K_M_ (catalytic efficiency) values were compared. PTE‐S5 has a catalytic efficiency of 60±10 M^−1^ s^−1^ against VX and 60±10 M^−1^ s^−1^ against VR. The three new variants outperformed PTE‐S5 against both VX and VR. PTE‐D1 performed with nearly five‐fold better efficiency against VX with a catalytic efficiency of 260±50 M^−1^ s^−1^ against VX but only two‐fold improvement to 150±10 M^−1^ s^−1^ against VR. In comparison, similar mutant studied by Cherny *et al*. possessed the active site mutation F132E as well as additional active site mutation T132N, which possessed 500‐fold and 5000‐fold increases in activity compared to S5.^[6]^ This comparison indicates the greater importance of T132N over F132E for PTE activity. For VR, PTE‐D2 and PTE‐D5 both showed three‐fold better efficiency against VX with catalytic efficiencies of 150±10 M^−1^ s^−1^ and 170±10 M^−1^ s^−1^ and over five‐fold improvement against VR with catalytic efficiencies of 260±40 M^−1^ s^−1^ and 360±10 M^−1^ s^−1^ respectively. In comparison, previous groups have generated PTE variants by targeting a larger subset of mutations, which allowed for activities ranging from 10^2^–10^8^ M^−1^ s^−1^ for both VX and VR_._[^8^, ^17^] Most notably, Raushel and coworkers designed the L7ep3a which was capable of 10^5^ M^−1^ s^−1^ VX hydrolyiss activity using enzyme evolution *via* error‐prone PCR^[18]^ and Tawfik and coworkers designed the d1‐IVA1 variant capable of the 10^5^ M^−1^ s^−1^ VR hydrolyiss activity using a combination of recombination experiments and computaitonal design.^[19]^ Recently, Köhler *et al*. engineered mutants of PTE capable of 10^9^ M^−1^ s^−1^ against VX while also maintaining high activity against G agents.^[17]^

**Table 4:** Summary of OP hydrolysis kinetics including lysate assay results and apparent catalytic activity for variants with activity against VX and VR. At the maximum regulated concentration of nerve agent (1 mM), no evidence of saturation was detected, indicating that K_M_>[S]. The Michaelis‐Menten equation was thus reduced to v0=k_cat_[E][S]/K_M_ and used to calculate the apparent catalytic efficiency values shown.

	Lysate assay	VX k_cat_/k_M_ (M^−1^ s^−1^)	VR k_cat_/k_M_ (M^−1^ s^−1^)
PTE‐S5	+	60±10	60±10
PTE‐D1	+	260±50	150±10
PTE‐D2	+	150±10	260±40
PTE‐D3	−	−	−
PTE‐D4	−	−	−
PTE‐D5	+	170±10	360±10
PTE‐D6	−	−	−

### Relationship between Mutations and Function

PTE‐S5 and PTE‐D1 differ only by the mutations shared among all of the D‐series variants: G208D, F104A, K77A, A80V, H254G, and I274N. These mutations collectively improve the efficiency of PTE against VX and VR without significantly affecting the protein structure or stability. PTE‐D2 and PTE‐D5, which both have improved efficiency against VR, share the I106A mutation. The I106 residue is adjacent to the leaving group pocket so mutating I106 to A with a less bulky amino acid side chain allows for reduced steric hindrance and improved catalysis. As discussed, the F132E mutation also allows for improved catalysis consistent with previous work where its mutation may allow electrostatic interaction with the V‐agent dialkyl group.^[6]^ The F132E variant reveals a 1.4‐fold imporvement over D2 against VR suggesting that its mutation provides a slight increase in catalyis when combined with I106A. We note that previously, the F132 residue was considered to not affect VX and VR breakdown, however these variants were compared in combination with many other stabilizing and active site mutations that substationally boosted V‐agent breakdown. Both the F132 and L271 conformational changes have been identified as critical to the shape of the active site.[^20^, ^21^] Moreover, PTE‐D3, PTE‐D4, and PTE‐D6 all share the L271E mutation, which abolishes catalytic activity. The three variants that share this mutation all demonstrate a change in predicted α‐helical content, and PTE‐D6 exhibits a further change of structure suggested by the θ_222_/θ_208_ greater than 1. This suggests that the L271E mutation impacts structure in a way that prevents catalysis.

## Conclusions

Using computational strategies, we have designed and synthesized a series of phosphotriesterase variants, some of which have improved efficiency against VX and VR organophosphate nerve agents. The mutations G208D, F104A, K77A, A80V, H254G, and I274N improve catalysis without impacting the structure of the enzyme. Specific effects of several point mutations near the active site have also been determined: I106A improves catalysis of VR and L271E abolishes activity even though it was predicted to improve it. Furthermore, alongside other recent work with V‐series nerve agents,[^6^, ^8^] we are able to carefully identify the relative contribution of point mutations I106A and F132E which will contribute to the design of catalytic OP bioscavengers with improved efficiency.

### Disclaimers

The views expressed in this manuscript are those of the authors and do not reflect the official policy of the Department of Army, Department of Defense, or the U.S. Government. This research was supported in part by an appointment to the Department of Defense (DOD) Research Participation Program administered by the Oak Ridge Institute for Science and Education (ORISE) through an interagency agreement between the U.S. Department of Energy (DOE) and the DOD. ORISE is managed by ORAU under DOE contract number DE‐SC0014664. All opinions expressed in this paper are the author‘s and do not necessarily reflect the policies and views of DOD, DOE, or ORAU/ORISE.

## Conflict of interests

The authors declare no conflict of interest.

1

## Supporting information

As a service to our authors and readers, this journal provides supporting information supplied by the authors. Such materials are peer reviewed and may be re‐organized for online delivery, but are not copy‐edited or typeset. Technical support issues arising from supporting information (other than missing files) should be addressed to the authors.

Supporting Information

## Data Availability

The data that support the findings of this study are available from the corresponding author upon reasonable request.
