# Supplementary material for: Computational Design of Phosphotriesterase Improves V‐Agent Degradation Efficiency
Source: ChemistryOpen. 2024 Mar 1;13(7):e202300263. doi: 10.1002/open.202300263 (PMC11230934; doi:10.1002/open.202300263)
Supplement: Supplementary file 1 — Supporting Information [file OPEN-13-e202300263-s001.pdf]

ChemistryOpen

Supporting Information

Computational Design of Phosphotriesterase Improves V-Agent Degradation Efficiency

Jacob Kronenberg, Stanley Chu, Andrew Olsen, Dustin Britton, Leif Halvorsen, Shengbo Guo, Ashwitha Lakshmi, Jason Chen, Maria Jinu Kulapurathazhe, Cetara A. Baker, Benjamin C. Wadsworth, Cynthia J. Van Acker, John G. Lehman, III, Tamara C. Otto, P. Douglas Renfrew, Richard Bonneau, and Jin Kim Montclare*

Computational Design of Phosphotriesterase Improves V-Agent Degradation Efficiency

Jacob Kronenberg[a], Stanley Chu[a], Andrew Olsen[a], Dustin Britton[a], Leif Halvorsen[bc], Shengbo Guo[a], Ashwitha Lakshmi[a], Jason Chen[a], Maria Jinu Kulapurathazhe[a], Cetara A. Baker[d], Benjamin C. Wadsworth[d], Cynthia J. Van Acker[d], John G. Lehman III[d], Tamara C. Otto[d], P. Douglas Renfrew[bc], Richard Bonneau[bc], Jin Kim Montclare*[aefgh]

[a] Department of Chemical and Biomolecular Engineering
New York University Tandon School of Engineering
Brooklyn, New York, United States
[b] Center for Genomics and Systems Biology
New York University
New York, New York, United States
[c] Center for Computational Biology
Flatiron Institute
New York, New York, United States
[d] Medical Toxicology Research Division
U.S. Army Medical Research Institute of Chemical Defense
Aberdeen Proving Ground, Maryland, United States
[e] Department of Biomaterials
New York University College of Dentistry
New York, New York, United States
[f] Department of Radiology
New York University Grossman School of Medicine
New York, New York, United States
[g] Department of Biomedical Engineering
New York University Tandon School of Engineering
Brooklyn, New York, United States
[h] Department of Chemistry
New York University
New York, New York, United States

Supporting Information

Materials

Urea, imidazole, sodium phosphate dibasic, sodium phosphate monobasic, cobalt chloride, magnesium sulfate, calcium chloride, ammonium chloride, sodium chloride, potassium phosphate monobasic, tris HCl, ampicillin, chloramphenicol, thiamine, methanol, acetic acid, hydrochloric acid, and glucose were obtained from Fisher Scientific. HisPur Co-NTA resin, tryptic soy agar, isopropyl-β-thiogalactopyranoside (IPTG) and SnakeSkin 10 kDa MWCO dialysis tubing were obtained from Thermo Scientific. 30% acrylamide solution and gel electrophoresis equipment were obtained from BioRad. 5,5'-dithio-bis-(2-nitrobenzoic acid) was purchased from Sigma-Aldrich. OP nerve agents were obtained from the U.S. Army Combat Capabilities Development Command Chemical Biological Center (Aberdeen Proving Ground, MD).

Computational Design of PTE Variants

Models of VX and VR nerve agents were constructed in YASARA.[1] VX(S) and VR(S) hydrolysis transition states were modeled in ORCA.[2] Briefly, VX and VR were docked in a model of PTE-S5[3] and coordinates of the active site residues H55, H57, L169, H201, and D301, the Co^{2+} ions, the attacking hydroxide ion, and the substrates were copied to ORCA. The bond between the hydroxide and the central phosphorus of the substrate was shortened while the bond between the leaving group and the central phosphorus was lengthened. The clusters with the highest energy were taken to be the transition state.

A series of sites were selected for targeted design based on variants previously described as having improved stability or efficacy against VX, VR, or V-agent analogs.[4–7] Rosetta energy score was used to determine stability. Binding energies were calculated as the difference in score

between models with the substrate transition state bound and unbound. The top six variants were selected for wet lab testing.

Expression and Purification of PTE

Genes coding for variants D1 and D5 were ordered from Genewiz and ligated into a pQE-30 vector. Site directed mutagenesis (SDM) was used to construct genes for the remaining variants. PCR was run for 30 cycles with 30 s denaturing at 94°C, 30 s annealing at 55°C and 4 min extension at 72°C, followed by a final 10 min extension step. PCR products were analyzed by gel electrophoresis in a 1% agarose gel in 1x TAE buffer. D2 was constructed by using SDM to introduce the I106A mutation to D1 (forward primer: 5′-GATGTGTCGACTGCGGATGCGGGTCGCGATGTCAGTTTATTG-3′). D3 and D4 were constructed by using SDM to introduce the L271E mutation to D1 and D2 respectively (forward primer: 5′-GCGAGTGCATCAGCCGAGCTGGGCAACCGTTCGTG-3′). D6 was constructed by using SDM to introduce the F132E mutation (forward primer: 5′-CGGCGACCGGCTTGTGGGAGGACCCGCCACTTTCG-3′).

PTE variants were expressed and purified as previously described.[3,8] Briefly, *E. coli* cells were transformed with pQE-30 plasmid vectors containing genes coding for each variant and grown on tryptic soy agar (TSA) plates with 200 μg/mL chloramphenicol (Cam) and 34 μg/mL ampicillin (Amp) to select for successful transformants.[9] Colonies were picked and grown overnight at 37°C and 350 rpm in 10 mL complete M9 media (0.5 M Na_2HPO_4, 0.22 M KH_2PO_4, 0.08 M NaCl, and 0.18 M NH_4Cl) supplemented with 10 mg/L of each of the 20 canonical amino acids, 1 mM $MgSO_4$, 0.1 mM $CaCl_2$, 0.2% w/v glucose, 200 μg/mL Amp, 34 μg/mL Cam, and 35 μg/mL thiamine. A 400 mL expression culture of supplemented M9 media was inoculated from

the starter culture and grown at 37°C and 350 rpm until the optical density at 600 nm (OD_{600}) reached 1.0, at which point 1 mM of $CoCl_2$ was added along with 1 mM IPTG to induce protein expression. After 3 hours at 37°C and 350 rpm, the expression colonies were harvested by centrifugation at 4000 rpm at 4°C for 10 minutes in an Avanti J15 centrifuge and stored frozen at -80°C. Protein expression was confirmed using 12% sodium dodecyl sulfate polyacrylamide gel electrophoresis (SDS-PAGE).

For purification, cells were resuspended in buffer A (20 mM Tris-HCl, 500 mM NaCl, 1 mM $CoCl_2$, 20 mM imidazole, pH 8) and lysed by sonication for 2.5 min at 75% amplitude with a QSonica Q500 probe sonicator, then the lysate was clarified by centrifugation at 10,000 rpm for 45 minutes at 4°C using an Avanti J15 centrifuge. Protein samples were purified by gravity flow column chromatography with a Co-NTA resin column bed using increasing concentrations of elution buffer B (20 mM Tris-HCl, 500 mM NaCl, 1 mM $CoCl_2$, 500 mM imidazole, pH 8). SDS-PAGE was used to confirm the purity of the eluted protein samples. Samples found to be pure were collected and dialyzed against PTE working buffer (20 mM Na_2HPO_4, 1 mM $CoCl_2$, pH 8), then concentrated using 10 kDa MWCO centrifugal filters. Protein sample concentrations were determined by bicinchoninic acid assay and samples were diluted to their working concentrations.

Circular Dichroism

Circular dichroism (CD) spectra were taken with a JASCO J-815 spectropolarimeter. Protein samples were diluted to 10 μM in PTE working buffer and 400 μL sample was added to a quartz cuvette with a 1 mm path length. Wavelength scans were taken from 190 nm to 250 nm at temperature intervals of 5°C between 25°C and 85°C. Additionally, the ellipticity at 222 nm, which correlates to the α-helical fraction, was measured every 1°C. Spectra were converted to

mean residual ellipticity and smoothed using a Savitzky-Golay filter[10] as previously described.[11] Melting temperatures (T_m) were determined by fitting the ellipticity at 222 nm as a function of temperature to a sigmoidal curve with the inflection point considered to be the T_m. Wavelength scans at 25°C were analyzed using BeStSel to determine the contribution of α-helices, β-sheets and other secondary structural components to the overall spectra.[12]

Differential Scanning Calorimetry

Differential scanning calorimetry (DSC) was performed using a NanoDSC from TA Instruments. Protein samples were diluted to 0.4 mg/mL in PTE working buffer and run against a reference of PTE working buffer with no protein. Calorimetry was performed from 20°C to 80°C at a scan rate of 0.5°C/min. A scan with PTE working buffer in both the sample and reference loops was used as a baseline. The scans were fit to a Gaussian model in NanoAnalyze software from TA Instruments to determine the melting temperatures as previously reported.[3,8]

Enzyme Kinetics

All V-agent kinetics experiments were performed at the U. S. Army Medical Research Institute of Chemical Defense (Aberdeen Proving Ground, MD). First, lysate containing each enzyme was assayed for activity against racemic mixtures of VX and VR. Lysate was clarified by centrifugation at 10,000 rpm for 45 minutes, then it was incubated with 0.75 mM of either VX or VR along with 3 mM Ellman's reagent (5,5′-dithiobis-(2-nitrobenzoic acid)) in a 96-well microplate. Absorbance at 412 nm was measured to determine whether each V-agent had undergone any hydrolysis.[13]

PTE protein was purified from lysates with detectable V-agent hydrolysis and assayed for its kinetic activity. 10 nM of purified enzyme was incubated with either VX or VR ranging from 10 μM to 1 mM along with a molar excess of Ellman's reagent. Absorbance at 412 nm was measured at regular intervals to monitor the progress of the reaction over time as a function of substrate concentration. The data was fit to a reduced Michaelis-Menten model to determine k_{cat}/K_M. All kinetics experiments were performed in triplicate.

Protein Biosynthesis

SDS-PAGE gels confirmed protein overexpression and purification (**Fig. S1**). Lysate sampled from expression flasks for all variants after the addition of IPTG showed an additional band at 37 kDa, corresponding to the size of the PTE monomer. Post-purification gels have single bands at 37 kDa, confirming that metal affinity chromatography can isolate the proteins of interest.

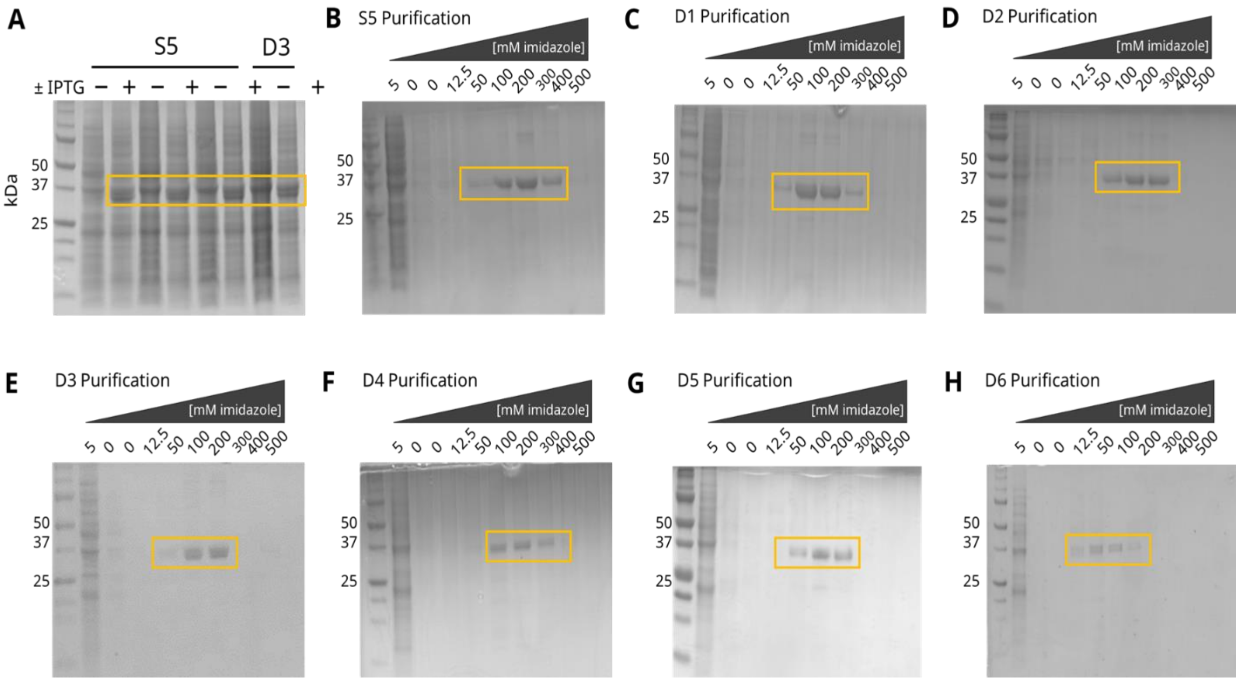

Figure S1. A. Representative SDS-PAGE gel of expression lysate samples before and after induction with IPTG showing expression of PTE-S5 and PTE-D3, as well as representative purification gels of B. PTE-S5, C. PTE-D1, D. PTE-D2, E. PTE-D3, F. PTE-D4, G. PTE-D5, H. PTE-D6.

Fluorination of PTE

Fluorination of PTE can improve its stability.[8,14] To investigate the effects of fluorination on the stability and kinetics of the variants described, PTE-S5 and PTE-D1 were fluorinated through global replacement of phenylalanine with *para*-fluorophenylalanine (pFF) as previously described to yield the fluorinated variants PTE-S5+pFF and PTE-D1+pFF.[8,14] Lysate assays were conducted on these

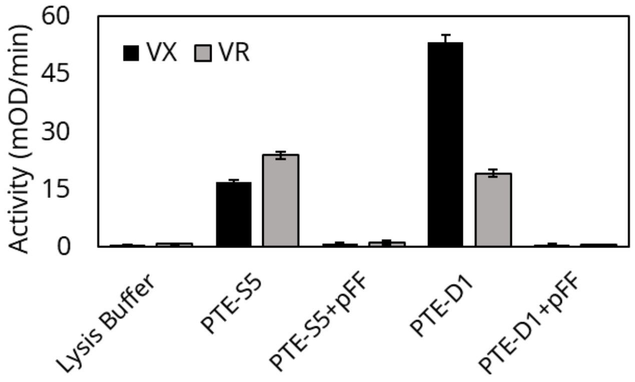

Figure S2. Activity as approximated by change in absorbance at 412 nm for reaction mixtures of VX and VR with lysis buffer as well as fluorinated and non-fluorinated PTE-S5 and PTE-D1.

fluorinated variants as described above (**Fig. S2**). Ultimately, the fluorinated variants tested showed no detectable catalytic activity, so further study of fluorinated PTE for VX and VR degradation was not pursued.

Works Cited

[1] E. Krieger, G. Vriend, *Bioinformatics* **2014**, *30*, 2981–2982.

[2] F. Neese, *Wiley Interdiscip. Rev. Comput. Mol. Sci.* **2012**, *2*, 73–78.

[3] A. J. Olsen, L. A. Halvorsen, C. Y. Yang, R. Barak Ventura, L. Yin, P. D. Renfrew, R. Bonneau, J. K. Montclare, *Mol. Biosyst.* **2017**, *13*, 2092–2106.

[4] I. Cherny, P. Greisen, Y. Ashani, S. D. Khare, G. Oberdorfer, H. Leader, D. Baker, D. S. Tawfik, *ACS Chem. Biol.* **2013**, *8*, 2394–2403.

[5] A. N. Bigley, C. Xu, T. J. Henderson, S. P. Harvey, F. M. Raushel, *J. Am. Chem. Soc.* **2013**, *135*, 10426–10432.

[6] A. N. Bigley, M. F. Mabanglo, S. P. Harvey, F. M. Raushel, *Biochemistry* **2015**, *54*, 5502–5512.

[7] L. Briseño-Roa, Z. Oliynyk, C. M. Timperley, A. D. Griffiths, A. R. Fersht, *Protein Eng. Des. Sel.* **2011**, *24*, 209–211.

[8] C. Y. Yang, P. D. Renfrew, A. J. Olsen, M. Zhang, C. Yuvienco, R. Bonneau, J. K. Montclare, *ChemBioChem* **2014**, *15*, 1761–1764.

[9] N. Sharma, R. Furter, P. Kast, D. A. Tirrell, *FEBS Lett.* **2000**, *467*, 37–40.

[10] M. J. E. Savitzky, A.; Golay, *Anal. Chem* **1964**, *36*, 1627–1639.

[11] S. K. Gunasekar, M. Asnani, C. Limbad, J. S. Haghpanah, W. Hom, H. Barra, S. Nanda,

M. Lu, J. K. Montclare, *Biochemistry* **2009**, *48*, 8559–8567.

[12] A. Micsonai, F. Wien, É. Bulyáki, J. Kun, É. Moussong, Y. H. Lee, Y. Goto, M. Réfrégiers, J. Kardos, *Nucleic Acids Res.* **2018**, *46*, W315–W322.

[13] T. C. Otto, C. K. Harsch, D. T. Yeung, T. J. Magliery, D. M. Cerasoli, D. E. Lenz, *Biochemistry* **2009**, *48*, 10416–10422.

[14] P. J. Baker, J. K. Montclare, *ChemBioChem* **2011**, *12*, 1845–1848.
